# The ferredoxin/thioredoxin pathway constitutes an indispensable redox-signaling cascade for light-dependent reduction of chloroplast stromal proteins

**DOI:** 10.1016/j.jbc.2022.102650

**Published:** 2022-11-29

**Authors:** Keisuke Yoshida, Yuichi Yokochi, Kan Tanaka, Toru Hisabori

**Affiliations:** Laboratory for Chemistry and Life Science, Institute of Innovative Research, Tokyo Institute of Technology, Yokohama, Japan

**Keywords:** *Arabidopsis thaliana*, CRISPR/Cas9, ferredoxin–thioredoxin reductase, redox regulation, thioredoxin, CF_1_-γATP synthase, CF_1_-γ subunit, 2-Cys Prx, 2-Cys peroxiredoxin, FBPase, fructose 1,6-bisphosphatase, Fd, ferredoxin, FTR, Fd–Trx reductase, FTRc, FTR catalytic subunit, GAPDH, glyceraldehyde 3-phosphate dehydrogenase, MDH, NADP-malate dehydrogenase, NTRC, NADPH–Trx reductase C, PRK, phosphoribulokinase, RCA, Rubisco activase, SBPase, sedoheptulose 1,7-bisphosphatase, Trx, thioredoxin

## Abstract

To ensure efficient photosynthesis, chloroplast proteins need to be flexibly regulated under fluctuating light conditions. Thiol-based redox regulation plays a key role in reductively activating several chloroplast proteins in a light-dependent manner. The ferredoxin (Fd)/thioredoxin (Trx) pathway has long been recognized as the machinery that transfers reducing power generated by photosynthetic electron transport reactions to redox-sensitive target proteins; however, its biological importance remains unclear, because the complete disruption of the Fd/Trx pathway in plants has been unsuccessful to date. Especially, recent identifications of multiple redox-related factors in chloroplasts, as represented by the NADPH–Trx reductase C, have raised a controversial proposal that other redox pathways work redundantly with the Fd/Trx pathway. To address these issues directly, we used CRISPR/Cas9 gene editing to create *Arabidopsis* mutant plants in which the activity of the Fd/Trx pathway was completely defective. The mutants generated showed severe growth inhibition. Importantly, these mutants almost entirely lost the ability to reduce several redox-sensitive proteins in chloroplast stroma, including four Calvin–Benson cycle enzymes, NADP–malate dehydrogenase, and Rubisco activase, under light conditions. These striking phenotypes were further accompanied by abnormally developed chloroplasts and a drastic decline in photosynthetic efficiency. These results indicate that the Fd/Trx pathway is indispensable for the light-responsive activation of diverse stromal proteins and photoautotrophic growth of plants. Our data also suggest that the ATP synthase is exceptionally reduced by other pathways in a redundant manner. This study provides an important insight into how the chloroplast redox-regulatory system operates *in vivo*.

Thiol-based redox regulation is a post-translational modification that controls protein function by switching the oxidation/reduction states of Cys residues (*e.g.*, formation/cleavage of disulfide bonds). As a mediator of reducing power, the small ubiquitous protein thioredoxin (Trx) plays a pivotal role in redox regulation. Trx contains a highly conserved amino acid sequence WCGPC at its active site. Using the two Cys residues in this sequence, Trx catalyzes a dithiol–disulfide exchange reaction with its target proteins, allowing their activities to be modulated. Trx is thus key to sensing local redox environments and tuning cell physiology accordingly (1, 2).

Trx-mediated redox regulation has important implication for plants that live under fluctuating light conditions. Although this regulatory system is preserved in all organisms, its mode of action in plant chloroplasts is unique in terms of being linked with light (3, 4). The thylakoid membrane converts light energy to reducing power through a series of photosynthetic electron transport reactions. Trx receives some of the reducing power from photosynthetically reduced ferredoxin (Fd) *via* Fd–Trx reductase (FTR) and then transfers it to several Trx-targeted proteins. In most cases, these Trx-targeted proteins are switched from inactive to active forms upon the reductive cleavage of specific disulfide bonds. Therefore, the redox cascade *via* the Fd/Trx pathway allows the activation of chloroplast proteins in concert with the excitation of photosynthetic electron transport and, thereby, light availability. The Fd/Trx pathway was identified about half a century ago and, since then, has been recognized as the hallmark of the redox-regulatory system in chloroplasts (3, 4). It is known that diverse chloroplast enzymes, including four Calvin–Benson cycle enzymes (Glyceraldehyde 3-phosphate dehydrogenase [GAPDH], fructose 1,6-bisphosphatase [FBPase], sedoheptulose 1,7-bisphosphatase [SBPase], and phosphoribulokinase [PRK]), ATP synthase, NADP–malate dehydrogenase (MDH), and Rubisco activase (RCA), are subject to redox regulation (3, 4, 5, 6, 7, 8, 9).

Owing to plant genomic and phylogenic studies, information about the factors constituting the redox-regulatory system in chloroplasts has been expanded greatly over the last 2 decades (10, 11, 12). In *Arabidopsis thaliana*, 10 Trx isoforms are targeted to chloroplasts, which are classified into five subtypes (Trx-*f*, Trx-*m*, Trx-*x*, Trx-*y*, and Trx-*z*). Each Trx subtype has a different redox potential and protein surface charge (13, 14, 15, 16, 17), resulting in their divergent functions (see later). In addition, several proteins containing putative redox-related motifs (*e.g.*, an atypical Trx active site sequence) have been identified in chloroplasts. NADPH–Trx reductase C (NTRC) is the most well-known example; this protein harbors both an NADPH–Trx reductase domain and a Trx domain in a single polypeptide and can use NADPH directly for redox regulation (18, 19). These advances have raised the hypothesis that plants have acquired a complex redox network in chloroplasts, enabling the flexible and sophisticated regulation of chloroplast functions. In fact, biochemical and reverse-genetic studies have revealed novel aspects of chloroplast redox regulation. The five Trx subtypes have been reported to exert specificity and redundancy in target recognition. Trx-*f* and Trx-*m* are largely responsible for the regulation of several metabolic enzymes and ATP synthase, whereas Trx-*x* and Trx-*y* are involved in the antioxidant defense system (13, 16, 20, 21, 22, 23, 24, 25). Trx-*z* plays a specific role in plastid gene expression as a component of plastid-encoded RNA polymerase complexes (26). Some Trx-like proteins designated as Trx-like2 and atypical Cys His-rich Trx were recently shown to mediate protein oxidation during light to dark transitions (27, 28, 29, 30, 31). Furthermore, NTRC has been suggested to regulate the 2-Cys peroxiredoxin (2-Cys Prx) redox balance (32, 33, 34), tetrapyrrole metabolism (33, 35, 36), and starch synthesis (37, 38). Despite these extensive studies, the whole picture of the chloroplast redox network and its biological significance is still unclear (39, 40, 41, 42, 43, 44, 45, 46, 47).

One fundamental but long-standing issue is to what extent the canonical Fd/Trx pathway is important to plants. As mentioned above, it has been generally accepted that the Fd/Trx pathway serves to activate various chloroplast enzymes in light (3, 4); however, this concept has been established mainly by *in vitro* reconstitution experiments, and its physiological relevance remains largely elusive. In particular, given the diversity of redox-related factors in chloroplasts, we must consider the possibility that other redox pathways may also participate in light-dependent redox regulation, thereby complementing the function of the Fd/Trx pathway. Indeed, NTRC was recently suggested to have an ability to regulate Calvin–Benson cycle enzymes and ATP synthase (42, 48, 49, 50, 51, 52). It is also possible that the glutathione/glutaredoxin pathway is involved in the regulation of these enzymes, because some of them were also identified as the glutaredoxin-interacting partners (53). Revisiting the role of the Fd/Trx pathway is, therefore, key to better understanding the redox-regulatory system in chloroplasts. In this study, we have addressed this issue by taking advantage of gene editing technology.

## Results

### Creation of FTR knockout mutants by CRISPR/Cas9 gene editing in *Arabidopsis*

**Figure 1:**
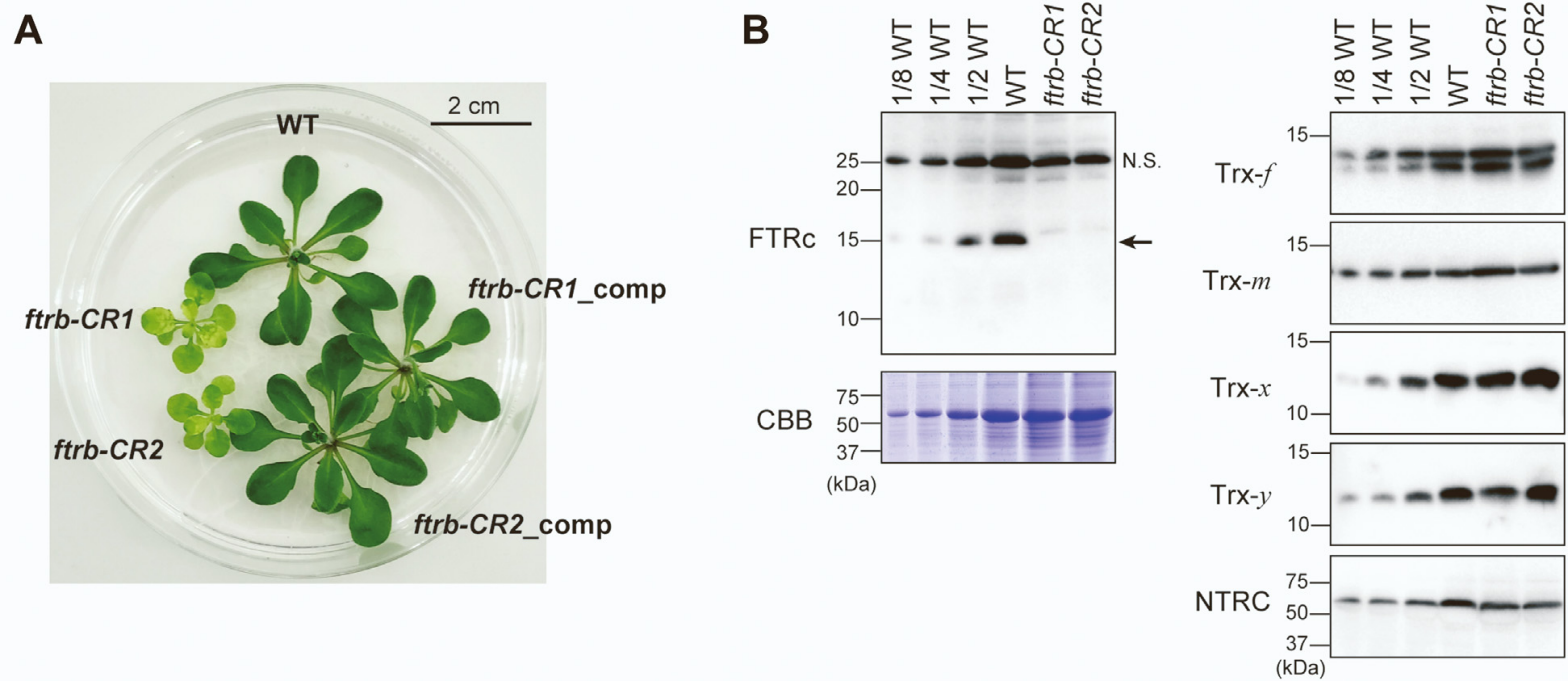
**Growth phenotypes of the *ftrb-CR* mutants.***A*, WT, *ftrb-CR* mutants, and *ftrb-CR*_comp plants were grown in half-strength Murashige and Skoog medium containing 2% (w/v) sucrose for 21 days. *B*, immunoblotting analyses of redox-related proteins. The same amount of total leaf protein (except for the WT dilution series) was loaded onto each lane. As a loading control, the Rubisco large subunit was stained with Coomassie Brilliant Blue R-250 (CBB). Experiments were performed using four different sample preparations, and representative results are shown. The *arrow* indicates the band for FTRc. NS indicates a possible nonspecific band.

FTR is a heterodimer protein composed of a catalytic subunit (FTRc) and a variable subunit. FTRc contains a [4Fe–4S] cluster and a redox-active disulfide bond, both of which are essential to catalysis (54). FTR can transfer Fd-derived reducing power to all the Trx isoforms in chloroplasts (17); therefore, FTR acts as a critical signaling hub in the Fd/Trx pathway. In addition, unlike plastid-targeted Trx, which is encoded by a total of 10 genes, FTRc is encoded by a single gene (*FTRB*) in *Arabidopsis*. Based on these facts, we focused on FTR as a target for disrupting the Fd/Trx pathway. Using CRISPR/Cas9 gene editing, we introduced a point mutation into the third exon of the *FTRB* gene in *Arabidopsis* (Fig. S1*A*). Consequently, we were able to isolate two types of mutants, designated as *ftrb-CR1* and *ftrb-CR2* (“*CR*” refers to CRISPR/Cas9). The *ftrb-CR1* mutant contained a T insertion, whereas the *ftrb-CR2* mutant contained a TCAT deletion (Fig. S1*B*). In both cases, a frameshift mutation was caused, leading to disruption of the *FTRB* gene. When grown in a sucrose-supplemented Murashige and Skoog medium, the *ftrb-CR* mutants showed severe growth phenotypes with pale-green leaves (Fig. 1*A*). The fresh weight of the aerial parts and leaf chlorophyll content were largely lowered in the *ftrb-CR* mutants (Fig. S2). We confirmed by immunoblotting analyses that the FTRc protein was undetectable in the *ftrb-CR* mutants (Figs. 1*B* and S3). On the other hand, other redox-related proteins, including the Trx subtypes and NTRC, did not show large changes in their accumulation levels (Fig. 1*B*). When grown in soil, the growth phenotypes of the *ftrb-CR* mutants became more marked (Fig. S4). These results indicate that FTR is an essential factor in plant autotrophic growth.

To check the impact of FTR deficiency on the growth phenotype, we transformed the wildtype *FTRB* gene under the control of the cauliflower mosaic virus *35S* promoter into the *ftrb-CR* mutant background. In these *FTRB*-complemented plants (*ftrb-CR1*_comp and *ftrb-CR2*_comp), the mutations in the intrinsic *FTRB* gene were maintained, but the FTRc protein accumulated because of the exogenously introduced *FTRB* gene (Figs. S1*B* and S3). The growth phenotypes were completely recovered in the *ftrb-CR*_comp plants (Figs. 1*A*, S2 and S4), confirming that FTR deficiency is responsible for growth inhibition in the *ftrb-CR* mutants.

### FTR is essential for light-dependent reduction of chloroplast stromal proteins

**Figure 2:**
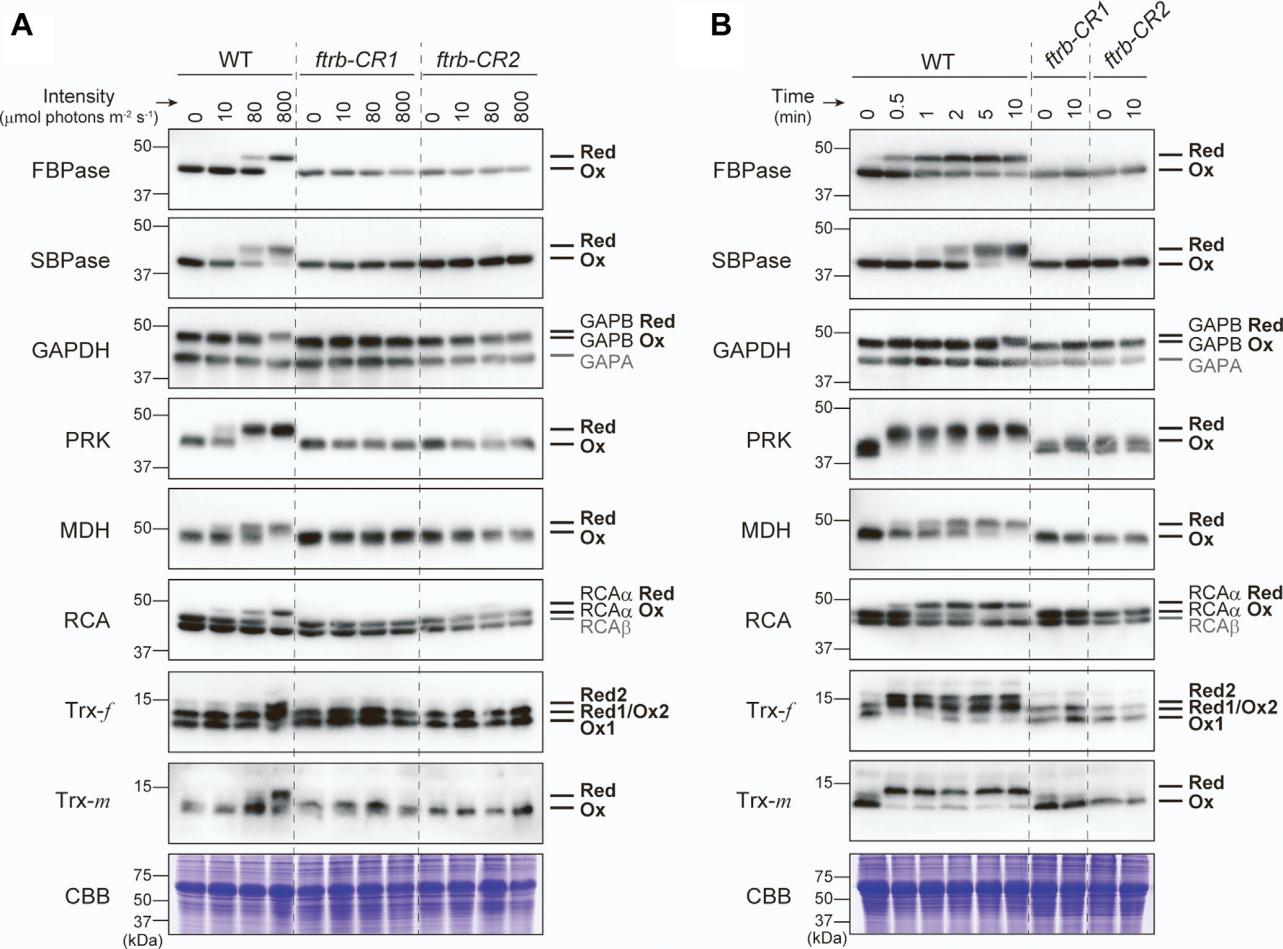
**Protein redox dynamics of stromal enzymes, Trx-*f* and Trx-*m* in WT and *ftrb-CR* mutants.***A*, plants were irradiated at the indicated light intensities for 20 min. *B*, plants were irradiated at 800 μmol photons m^−2^ s^−1^ for the period indicated. The same amount of total leaf protein was loaded onto each lane. As a loading control, the Rubisco large subunit was stained with Coomassie Brilliant Blue R-250 (CBB). For GAPDH, redox-sensitive GAPB and redox-insensitive GAPA were detected. For RCA, redox-sensitive RCAα and redox-insensitive RCAβ were detected. Experiments were performed using three different sample preparations, and representative results are shown. Ox, oxidized form; Red, reduced form.

We analyzed the light-responsive changes in the protein redox states using a thiol-modifying reagent (55). Wildtype and *ftrb-CR* mutant plants were irradiated at several light intensities (0, 10, 80, and 800 μmol photons m^−2^ s^−1^) (Fig. 2*A*). In the wildtype plants, four Calvin–Benson cycle enzymes, including FBPase, SBPase, GAPDH (redox-sensitive GAPB isoform), and PRK, were shifted from the oxidized to reduced forms in response to increased light intensity. Similarly, MDH and RCA (redox-sensitive RCAα isoform) were reduced in a light-dependent manner. By contrast, these stromal proteins could not be reduced under any light conditions in the *ftrb-CR* mutants. We then investigated the time course of the protein redox states after irradiation (Fig. 2*B*). We have previously demonstrated the different reduction kinetics of FBPase, SBPase, and RCA (56). The present study clarified the distinct redox responses more comprehensively. SBPase and GAPDH showed relatively slow reduction patterns, whereas PRK rapidly reached a fully reduced state; FBPase, MDH, and RCA were reduced at intermediate rates. Recently, Zimmer *et al.* (57) reported the protein redox responses during dark to light transitions at the proteome level; our present data (*e.g.*, rapid reduction of PRK) appear to be in line with their observations and further highlight the dynamics of chloroplast redox regulation in light. These redox responses were not detected in the *ftrb-CR* mutants, indicating that FTR is essential for the light-dependent reductive activation of various stromal proteins.

We also assessed the redox states of Trx-*f* and Trx-*m*. It is known that these Trx subtypes are mainly involved in the activation of several metabolic enzymes (*e.g.*, Calvin–Benson cycle enzymes) (13, 16). In the wildtype plants, both Trx-*f* and Trx-*m* were reduced under strong light conditions (800 μmol photons m^−2^ s^−1^) (Fig. 2*A*). They were apparently present in the oxidized states under weak (10 μmol photons m^−2^ s^−1^) and growth (80 μmol photons m^−2^ s^−1^) light conditions, which was possibly because of higher rates of oxidation by their target proteins than those of reduction. Trx reduction responses were quite rapid; both Trx-*f* and Trx-*m* reached stably reduced states within 30 s after irradiation (Fig. 2*B*). In the *ftrb-CR* mutants, neither Trx-*f* nor Trx-*m* was reduced under any light conditions. These results indicate that FTR acts as the major transmitter of reducing power to Trx-*f* and Trx-*m* in light. In the *ftrb-CR*_comp plants, the light-dependent reduction of stromal proteins, Trx-*f* and Trx-*m*, was observed as in the wildtype plants (Fig. S5).

### FTR is essential for photosynthesis and chloroplast development

**Figure 3:**
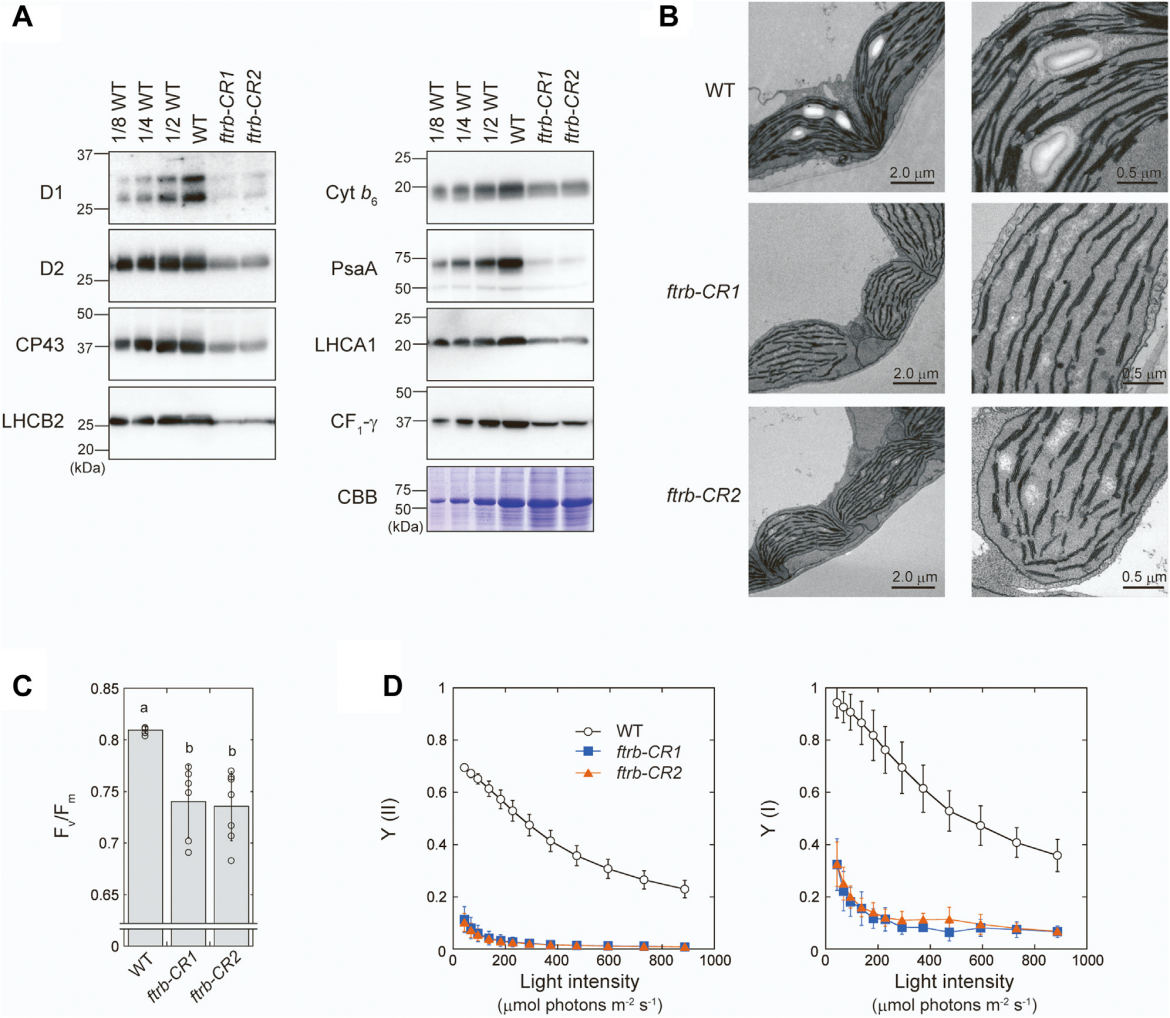
**Photosynthesis-related physiological traits of *ftrb-CR* mutants.***A*, immunoblotting analyses of photosynthetic proteins. The same amount of total leaf protein (except for the WT dilution series) was loaded onto each lane. As a loading control, the Rubisco large subunit was stained with Coomassie Brilliant Blue R-250 (CBB). Experiments were performed using at least three different sample preparations, and representative results are shown. *B*, chloroplast ultrastructure as observed by electron microscopy. Data were collected using approximately 10 leaf segments, and representative results are shown. *C* and *D*, photosynthetic electron transport measurements. *C*, maximal photochemical quantum yield of photosystem II (F_v_/F_m_). *D*, operating efficiencies of photosystem II [Y (II)] and photosystem I [Y (I)]. Y (II) and Y (I) were determined at several light intensities. Each value represents the mean ± SD (n = 6–7 biological replicates). The different *letters* in (*C*) indicate significant differences (*p* < 0.05, Tukey–Kramer multiple comparison test).

We investigated several physiological traits related to photosynthesis. The accumulation of several photosynthetic proteins (electron transport proteins and light-harvesting proteins) was drastically lowered in the *ftrb-CR* mutants (Fig. 3*A*). The observation of chloroplast ultrastructure using electron microscopy showed that, in contrast to the wildtype plants that contained well-organized thylakoid membranes, the *ftrb-CR* mutants had less stacked and poorly developed thylakoid membranes (Fig. 3*B*). Moreover, starch granules were not observed in the *ftrb-CR* mutants. Chlorophyll fluorescence measurements showed lower F_v_/F_m_ values in the *ftrb-CR* mutants (Fig. 3*C*), indicating that these mutants are more susceptible to photosystem II photoinhibition. We further evaluated photosynthetic electron transport property by monitoring the chlorophyll fluorescence and P700 absorbance changes at several light intensities (Figs. 3*D* and S6). The operating efficiencies of photosystem II and photosystem I were much lower in the *ftrb-CR* mutants under all light conditions examined (Fig. 3*D*). Several parameters related to electron transport were largely affected in the *ftrb-CR* mutants, as reflected by higher nonphotochemical quenching [Y (NPQ)] and P700 acceptor-side limitation [Y (NA)] (Fig. S6). Taken together, these results indicate that FTR is essential for optimal photosynthetic performance and chloroplast development.

### FTR is not essential for light-dependent reduction of ATP synthase CF_1_-γ subunit

**Figure 4:**
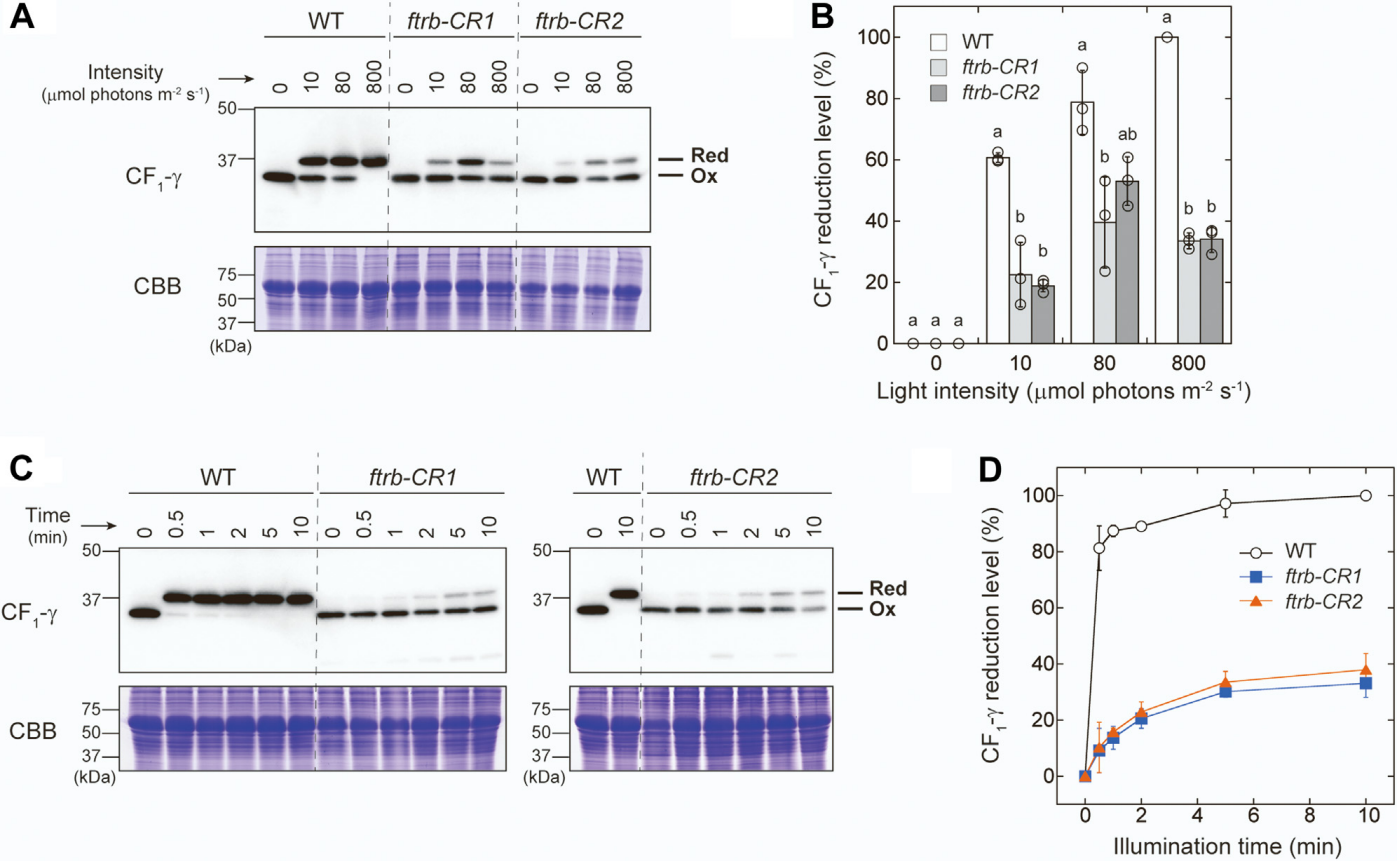
**Protein redox dynamics of ATP synthase CF**_**1**_**-γ subunit in WT and *ftrb-CR* mutants.***A* and *B*, plants were irradiated at the indicated light intensities for 20 min. *C* and *D*, plants were irradiated at 800 μmol photons m^−2^ s^−1^ for the period indicated. *A* and *C*, the same amount of total leaf protein was loaded onto each lane. As a loading control, the Rubisco large subunit was stained with Coomassie Brilliant Blue R-250 (CBB). *B* and *D*, the CF_1_-γ reduction level was calculated as the ratio of the reduced form to the total. Each value represents the mean ± SD (n = 3 biological replicates). The different *letters* indicate significant differences (*p* < 0.05, Tukey–Kramer multiple comparison test). Ox, oxidized form; Red, reduced form.

We analyzed the light-responsive redox changes of the ATP synthase at the thylakoid membrane. In this protein complex, the CF_1_-γ subunit (CF_1_-γ) contains two Cys residues that are targets for thiol modulation and is thus key to the redox regulation of ATP synthase (6, 58). In the wildtype plants, CF_1_-γ was sensitively reduced even under weak light conditions (Fig. 4, *A* and *B*). CF_1_-γ was also rapidly reduced in response to light; its reduction level reached more than 80% within 30 s after irradiation (Fig. 4, *C* and *D*). Notably, even in the *ftrb-CR* mutants, CF_1_-γ could be reduced upon illumination, although its reduction efficiency was lower than that seen in the wildtype plants (Fig. 4). It has been generally considered that CF_1_-γ is reduced by the Fd/Trx pathway in light (6, 58). However, our results suggest that a different redox pathway also serves to transfer reducing power to CF_1_-γ.

**Figure 5:**
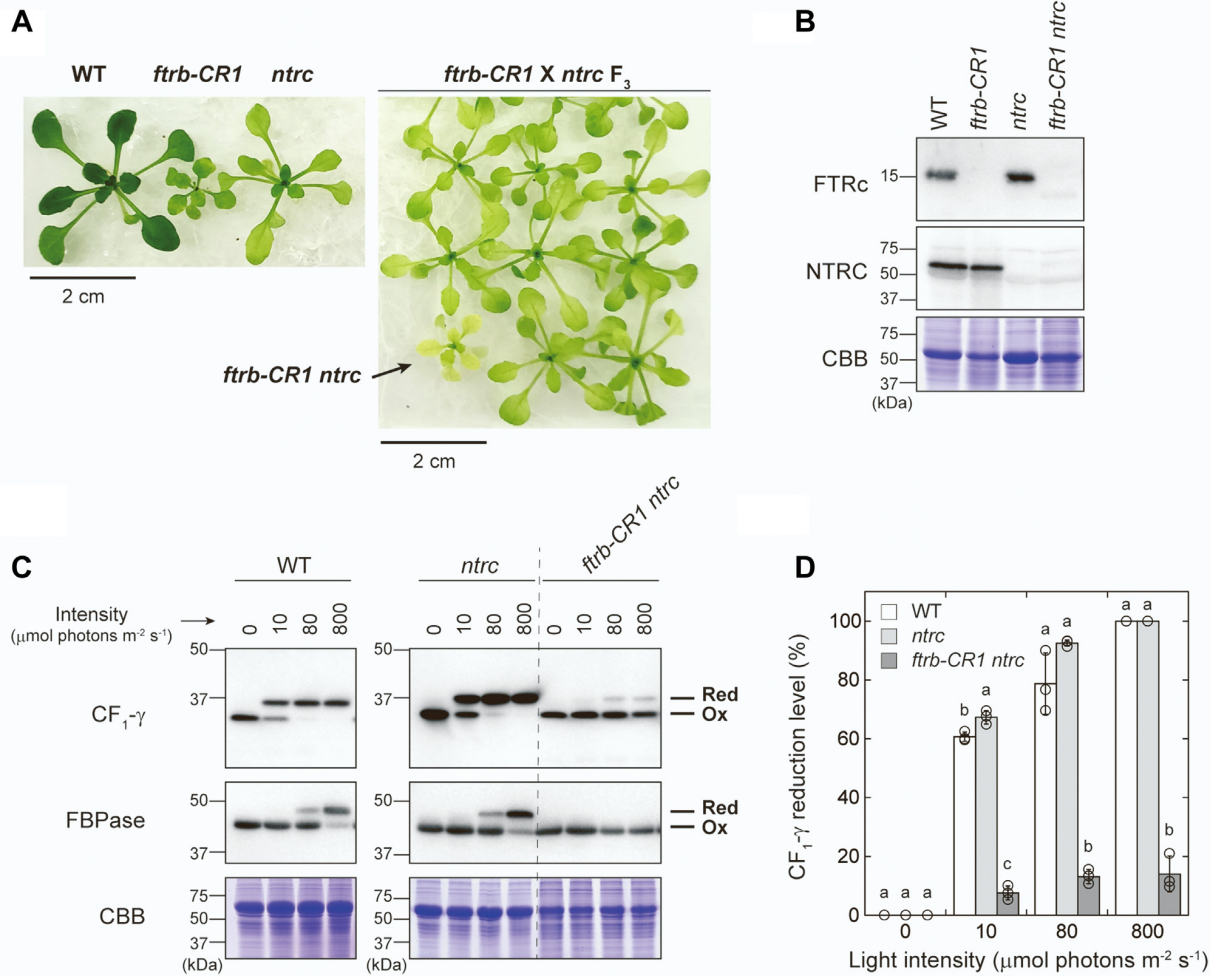
**Effect of simultaneous defects of FTRc and NTRC on redox regulation of ATP synthase CF**_**1**_**-γ subunit.***A*, generation of the *ftrb-CR1 ntrc* double mutant. The *ftrb-CR1 ntrc* double homozygous mutant (indicated by an *arrow*) was isolated from the F_3_ generation of *ftrb-CR1* × *ntrc* based on genomic PCR and DNA sequencing analysis (Fig. S7). Plants grown in half-strength Murashige and Skoog medium containing 2% (w/v) sucrose for 18 days are shown. *B*, immunoblotting analyses of FTRc and NTRC. The same amount of total leaf protein was loaded onto each lane. As a loading control, the Rubisco large subunit was stained with Coomassie Brilliant Blue R-250 (CBB). *C* and *D*, protein redox dynamics of CF_1_-γ and FBPase in the WT, *ntrc* mutant, and *ftrb-CR1 ntrc* double mutant. Plants were irradiated at the indicated light intensities for 20 min. *C*, as a loading control, the Rubisco large subunit was stained with CBB. *D*, the CF_1_-γ reduction level was calculated as the ratio of the reduced form to the total. Each value represents the mean ± SD (n = 3 biological replicates). The different *letters* indicate significant differences (*p* < 0.05, Tukey–Kramer multiple comparison test). Ox, oxidized form; Red, reduced form.

NTRC is a candidate for the alternative pathway because it uses NADPH as a reductant for redox regulation and is thus able to work independently from the Fd/Trx pathway (18, 19). In fact, previous studies have suggested that NTRC is involved in CF_1_-γ redox regulation (42, 49, 50, 51, 52). To address this possibility, we attempted to create mutant plants defective in both FTR and NTRC. We crossed the *ftrb-CR1* mutant with the NTRC-knockout T-DNA insertion mutant (the *ntrc* mutant) (33). Eventually, we isolated *ftrb-CR1 ntrc* double homozygous mutants from the F_3_ generation (Figs. 5*A* and S7; see the Experimental procedures section). The mutant plants obtained here were used directly for the next experiments because they were unable to produce progeny seeds because of extremely severe growth inhibition. We confirmed that the *ftrb-CR1 ntrc* mutant was defective in both FTRc and NTRC proteins (Fig. 5*B*). We then analyzed the light-responsive redox changes of CF_1_-γ. CF_1_-γ reduction efficiency in the *ntrc* single mutants was comparable with or even higher than that in the wildtype plants (Fig. 5, *C* and *D*). In the *ftrb-CR1 ntrc* mutants, CF_1_-γ reduction was largely impaired; however, more notably, CF_1_-γ could still be partially reduced. These results suggest that unlike stromal proteins, whose reduction is absolutely dependent on the Fd/Trx pathway, CF_1_-γ can be reduced by multiple redox pathways.

## Discussion

Extensive research has been made to elucidate the redox-regulatory system in chloroplasts, but a consistent view of this system is yet to be established (39, 40, 41, 42, 43, 44, 45, 46, 47). A key issue is that, despite the long period since its discovery, the biological importance of the Fd/Trx pathway remains elusive. Here, we report the indispensable role of the Fd/Trx pathway in chloroplast redox regulation.

To study the role of the Fd/Trx pathway, we noted that FTR is a central redox transmitter in this pathway. Some mutant plants of FTR have been reported previously in *Arabidopsis* (33, 59, 60, 61). Wang *et al.* (60) reported that the virus-induced gene silencing of the *FTRB* gene resulted in delayed leaf greening accompanied by alterations in plastid gene expression. We also reported two different strains of FTR mutants (33, 61). T-DNA insertion into the *FTRB* intron region lowered both the *FTRB* transcript and FTRc protein levels to 10 to 25% of those in wildtype plants, causing mild retardation of plant autotrophic growth (33). One amino acid substitution in the FTRc protein (Cys^60^ to Tyr), which modified the catalytic ability of FTR, led to changes in the metabolic profiles of the plants (61). Based on these phenotypes, we can, to some extent, infer the physiological role of FTR. However, being not complete knockout strains, these mutants were still weak to discuss the functional consequences of FTR *in vivo*. In addition, the direct impact of FTR mutation on redox regulation itself has been less characterized. These issues have hampered our understanding of the nature of the Fd/Trx pathway and the overall redox-regulatory system in chloroplasts.

**Figure 6:**
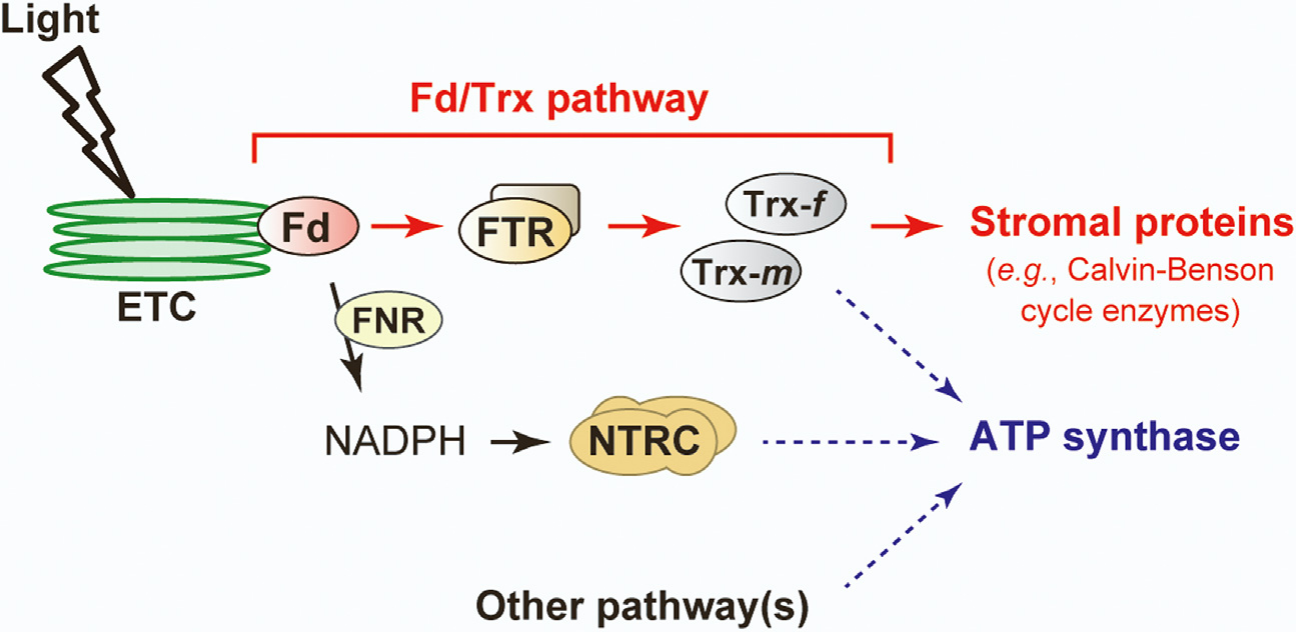
**Simplified model of chloroplast redox regulation in the light.** The *arrows* indicate the direction of reducing power transfer. See the main text for details. ETC, electron transport chain; FNR, Fd-NADP^+^ reductase.

To overcome these limitations, we created FTR-knockout *ftrb-CR* mutant plants using CRISPR/Cas9 gene editing (Figs. 1 and S1). The phenotypes of the *ftrb-CR* mutants underpinned the critical importance of the Fd/Trx pathway in photosynthesis and growth (Figs. 1, 3 and S2). By determining the protein redox states directly (55), we revealed that the light-dependent reduction of FBPase, SBPase, GAPDH, PRK, MDH, and RCA was almost entirely lost in the *ftrb-CR* mutants (Fig. 2). Their reducing factors, Trx-*f* and Trx-*m*, were also not reduced under any light conditions in the *ftrb-CR* mutants (Fig. 2). These results suggest in a straightforward manner that the Fd/Trx pathway is the sole pathway to transmit light-derived reductive signals and thereby activate a range of stromal redox-sensitive proteins in light (Fig. 6). On the other hand, previous studies showed that, in NTRC-overexpressing *Arabidopsis* plants, some Calvin–Benson cycle enzymes were present in the more reduced states (51, 52). In addition, interactions of NTRC with some Calvin–Benson cycle enzymes and Trx isoforms were shown using bimolecular fluorescence complementation and coimmunoprecipitation tests (51). Based on these observations, NTRC was suggested to mediate the redox regulation of the Calvin–Benson cycle (42, 51, 52). However, this suggestion is not supported by the data presented in this study. It should be noted that the inefficiency of NTRC in reducing Trx-*f*, Trx-*m*, and Calvin–Benson cycle enzymes was made evident by *in vitro* analyses using purified proteins (33, 62). Alternatively, a convincing model for NTRC function was recently proposed on the basis of the identification of *ntrc*-suppressor mutants (31, 34, 63); NTRC maintains the redox balance of the 2-Cys Prx pool, which is essential for optimal working of chloroplast redox regulation (45). It is thus reasonable to consider that NTRC is involved in the redox regulation of the Calvin–Benson cycle *via* an indirect mechanism.

In contrast to stromal proteins, ATP synthase CF_1_-γ subunit was substantially reduced under light conditions in the *ftrb-CR* mutants (Fig. 4). This redox response was partially retained even when NTRC was simultaneously disrupted (Fig. 5). These results suggest that the reductive activation of ATP synthase is potentially supported by multiple redox pathways, including the Fd/Trx pathway, the NTRC pathway, and a yet unidentified pathway (Fig. 6). This regulatory feature may enable rapid reduction of CF_1_-γ (56, 64, 65) and rapid activation of ATP synthase (66) after irradiation. At this stage, it is difficult to determine how much each pathway contributes to CF_1_-γ reduction. Strong phenotypes in the mutants (*e.g.*, pale-green leaves [Figs. 1*A* and 5*A*] and an impaired electron transport reaction [Fig. 3*D*]) prevent simple data interpretation. Hence, more extensive studies covering biochemical approaches are required to evaluate this issue.

The identification of the Fd/Trx pathway by Buchanan *et al.* (3, 4) about half a century ago represents a landmark advance in plant science; however, the biological importance of this pathway has been experimentally undetermined to date. Our present data allow us to conclude that the Fd/Trx pathway constitutes an indispensable redox-signaling cascade to regulate a range of stromal proteins under light conditions and support photosynthetic biomass production. We also found that other redox pathways may be involved in the regulation of ATP synthase in a redundant manner. This study provides a robust view of the *in vivo* working mechanism of the chloroplast redox-regulatory system and a perspective for elucidating this system more deeply.

## Experimental procedures

### Plant materials and growth conditions

*A. thaliana* Col-0 was used as the wildtype plant. The T-DNA-inserted *ntrc* mutant (Salk_114293C) was prepared previously (33). The *ftrb-CR* mutants (*ftrb-CR1* and *ftrb-CR2*), *ftrb-CR*_comp plants (*ftrb-CR1*_comp and *ftrb-CR2*_comp), and *ftrb-CR1 ntrc* double mutants were newly created as described later. Plants were grown in half-strength Murashige and Skoog medium supplemented with 2% (w/v) sucrose in a controlled growth chamber (80 μmol photons m^−2^ s^−1^, 22 °C, 16 h day/8 h night) for approximately 3 weeks and then used for the following experiments. Plants were also grown in soil in the same growth chamber (Fig. S4).

### Creation of *ftrb-CR* mutants using CRISPR/Cas9 gene editing

The CRISPR/Cas9-based creation of *ftrb-CR* mutants was performed according to Hahn *et al.* (67). A plasmid for CRISPR/Cas9 gene editing was constructed using the pFH6_new vector, pUB-Cas9 vector, and primers shown in Table S1. A single-guide RNA expression cassette was integrated into the plasmid using the primers FH41 and FH42 (Table S1). Wildtype plants were transformed with the resulting plasmid using the *Agrobacterium*-mediated floral dip method (68). The homozygous *ftrb-CR* mutants were screened based on DNA sequencing analysis.

### Complementation of *FTRB* gene in *ftrb-CR* mutants

The full-length *FTRB*-coding region was amplified using the primers shown in Table S1 and inserted into the pRI 201-AN vector (Takara). Plants were transformed with the resulting plasmid using the *Agrobacterium*-mediated floral dip method (68). Because it was difficult to use the homozygous *ftrb-CR* mutants because of their severe growth phenotypes, the heterozygous mutants were used for the transformation. Progeny plants containing the homozygous mutations in the intrinsic *FTRB* gene (as determined by DNA sequencing analysis) and accumulating the FTRc protein (as determined by immunoblotting analysis) were selected as the *ftrb-CR*_comp plants.

### Creation of *ftrb-CR1 ntrc* double mutant

The *ftrb-CR1 ntrc* double mutant was obtained by crossing *ftrb-CR1* and *ntrc* mutants. Although we could isolate the *ftrb-CR1 ntrc* double homozygous mutants from the F_2_ generation, they showed severe growth inhibition and were unable to produce progeny seeds. We therefore reisolated *ftrb-CR1* heterozygous/*ntrc* homozygous mutants from the F_2_ generation. This procedure enabled us to isolate the *ftrb-CR1 ntrc* double homozygous mutants efficiently from the following F_3_ generation. The plant genotypes were determined using genomic PCR with the primers listed in Table S1 (for *ntrc*) and DNA sequencing analysis (for *ftrb-CR1*).

### Chlorophyll content and a/b ratio

The chlorophyll content and a/b ratio were determined after extraction with 80% (v/v) acetone according to the method described (69).

### Protein accumulation

The accumulation of several redox-related proteins and photosynthetic proteins was examined by immunoblotting analysis. The total leaf protein was extracted as described previously (70). The proteins were separated by SDS-PAGE and transferred to a polyvinylidene difluoride membrane. Antibodies against FTRc, Trx-*f* (raised against Trx-*f*1 isoform), Trx-*m* (raised against Trx-*m*2 isoform), Trx-*x*, Trx-*y* (raised against Trx-*y*2 isoform), and NTRC were prepared previously (16, 17, 33, 65). For several photosynthetic proteins shown in Figure 3*A*, commercially available antibodies were used (Agrisera), except for the CF_1_-γ antibody, which was prepared previously (65).

### Determination of protein redox state

A detailed protocol to determine the protein redox state *in vivo* is available in our previous paper (55). In brief, plants were directly frozen using liquid nitrogen under the indicated conditions. The extracted proteins were labeled with the specific thiol-modifying reagent 4-acetamido-4′-maleimidylstilbene-2,2′-disulfonate (Invitrogen). This reagent has a molecular mass of 536.44 and thereby lowers protein mobility on SDS-PAGE, allowing the determination of the protein redox state from an observable band shift. Antibodies against FBPase, SBPase, PRK, MDH, and 2-Cys Prx were prepared previously (16, 31, 33, 65). GAPDH antibody was created using recombinant GAPB protein as the antigen. For RCA, commercially available antibody was used (Agrisera). For detecting Trx-*f*, Trx-*m*, and CF_1_-γ, the same antibodies as described in the *Protein accumulation* section were used.

### Photosynthetic electron transport

The chlorophyll fluorescence and P700 absorbance change around 830 nm were measured simultaneously using a Dual-PAM/F (Walz) with the intact leaves. A saturating pulse of red light (800 ms, >5000 μmol photons m^−2^ s^−1^) was applied to calculate several parameters. After measuring F_v_/F_m_ and the maximal P700 absorbance change in the dark-adapted state, actinic light (red light) was used to irradiate the leaves. The intensity of actinic light was elevated from low to high levels in a stepwise manner. The methods used to calculate the quantum yields of PSII [Y (II), Y (NO), and Y (nonphotochemical quenching)] and photosystem I [Y (I), Y (ND), and Y (NA)] were as described (71).

### Chloroplast ultrastructure

After 5 h of exposure to growth light, leaf samples were cut into small pieces and fixed in 1/15 M phosphate buffer (pH 7.4) containing 2% (w/v) paraformaldehyde and 2.5% (v/v) glutaraldehyde at 4 °C overnight. After washing with 1/15 M phosphate buffer (pH 7.4) five times, the samples were postfixed in 1/15 M phosphate buffer (pH 7.4) containing 2% (w/v) osmium tetroxide for 2 h. After washing with chilled 1/15 M phosphate buffer (pH 7.4) containing 8% (w/v) sucrose twice, the samples were dehydrated with an ethanol series (30–100%) and infiltrated with resin overnight. The samples were placed into molds filled with resin. Polymerization was performed at 60 °C for 2 days. Next, the samples were ultrasectioned and stained, and the chloroplast ultrastructure was observed using a transmission electron microscopy.

## Data availability

All data are contained within the article.

## Supporting information

This article contains supporting information.

## Conflict of interest

The authors declare that they have no conflicts of interest with the contents of this article.
