# Supplementary material for: The ferredoxin/thioredoxin pathway constitutes an indispensable redox-signaling cascade for light-dependent reduction of chloroplast stromal proteins
Source: J Biol Chem. 2022 Nov 29;298(12):102650. doi: 10.1016/j.jbc.2022.102650 (PMC9712825; doi:10.1016/j.jbc.2022.102650)
Supplement: Supplemental Figures S1–S7 and Table S1 [file mmc1.pdf]

Supporting Information for

The ferredoxin/thioredoxin pathway constitutes an indispensable redox-signaling cascade for light-dependent reduction of chloroplast stromal proteins

Keisuke Yoshida[1*], Yuichi Yokochi[1], Kan Tanaka[1], and Toru Hisabori[1]

[1]Laboratory for Chemistry and Life Science, Institute of Innovative Research, Tokyo Institute of Technology, Yokohama, Japan

[*]Corresponding Author: Keisuke Yoshida
Email: kyoshida@res.titech.ac.jp

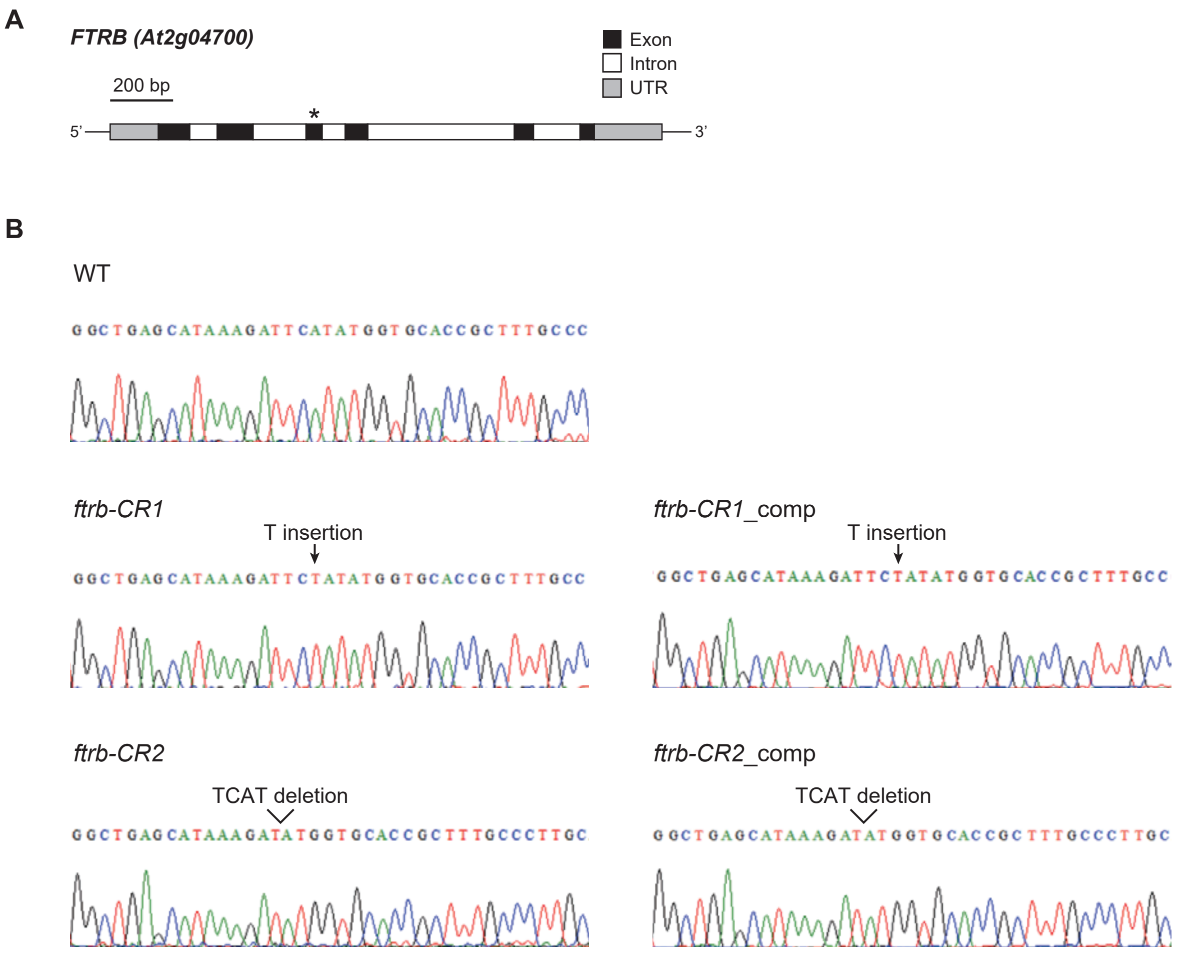

Fig. S1. CRISPR/Cas9-based construction of *ftrb-CR* mutants. (A) Simplified structure of the *FTRB* gene. A Cas9-targeted site is shown by the asterisk. (B) DNA sequences around the Cas9-targeted site of wild type (WT), *ftrb-CR* mutants, and *ftrb-CR*_comp plants.

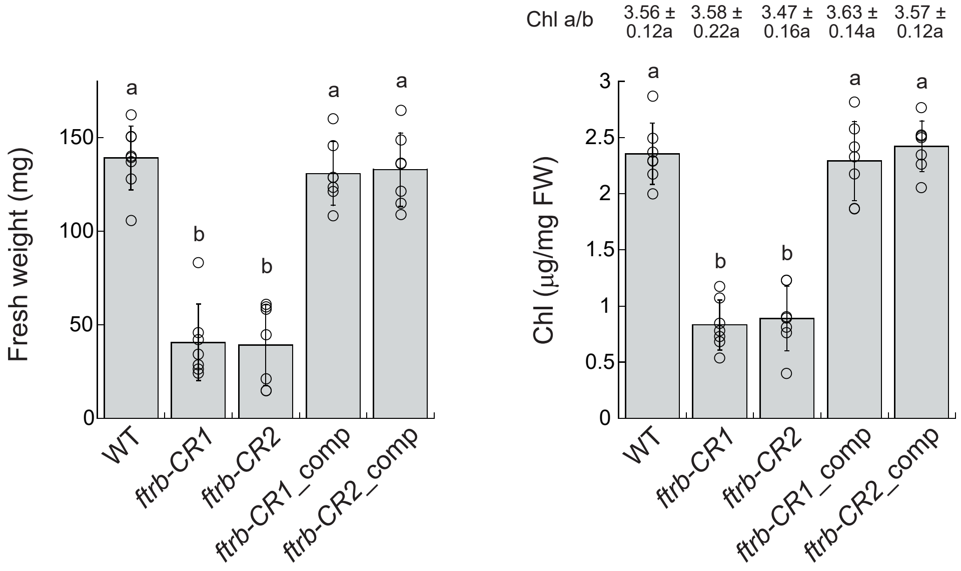

Fig. S2. Growth parameters of WT, *ftrb-CR* mutants, and *ftrb-CR*_comp plants. Fresh weight of aerial parts, chlorophyll (Chl) content, and Chl a/b ratio are shown. Each value represents the mean ± SD (n = 7–8 biological replicates). The different letters indicate significant differences ($P < 0.05$, Tukey-Kramer multiple comparison test).

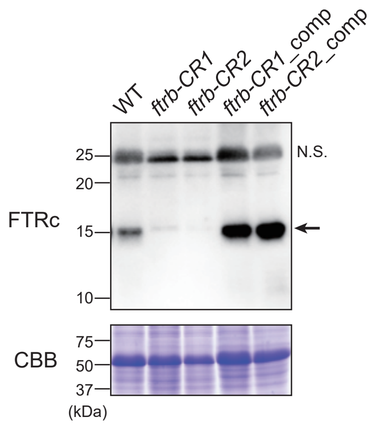

Fig. S3. Accumulation of FTRc protein in *ftrb-CR*_comp plants. The immunoblotting data for FTRc are shown. The same amount of total leaf protein was loaded onto each lane. As a loading control, the Rubisco large subunit was stained with Coomassie Brilliant Blue R-250 (CBB). The arrow indicates the band for FTRc. N.S. indicates a possible nonspecific band.

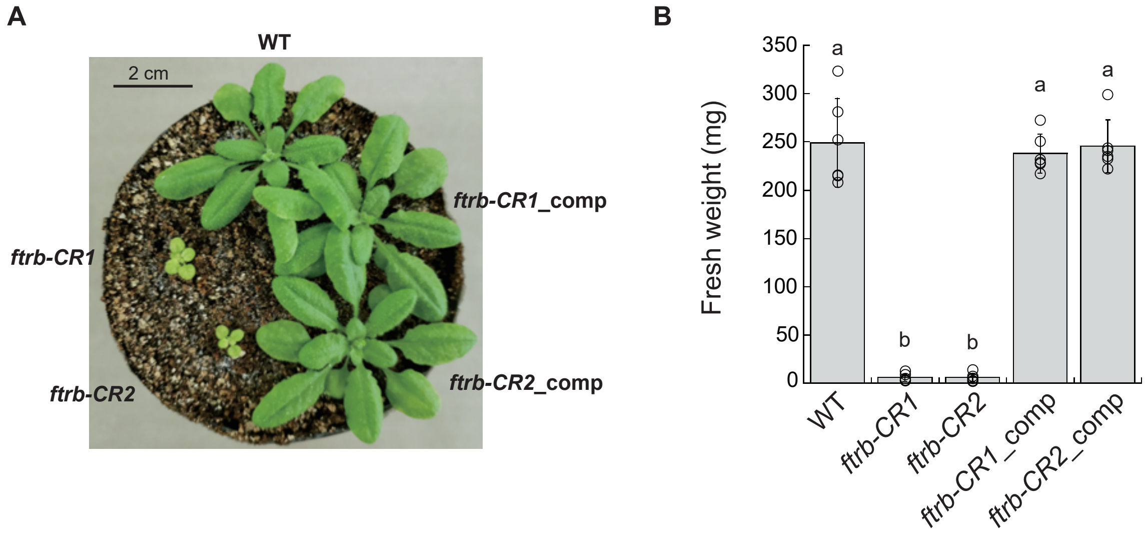

Fig. S4. Growth phenotypes of *ftrb-CR* mutants under autotrophic conditions. (A) WT, *ftrb-CR* mutants, and *ftrb-CR*_comp plants were grown in soil for 25 days. (B) Fresh weight of aerial parts. Each value represents the mean ± SD (n = 6 biological replicates). The different letters indicate significant differences ($P < 0.05$, Tukey-Kramer multiple comparison test).

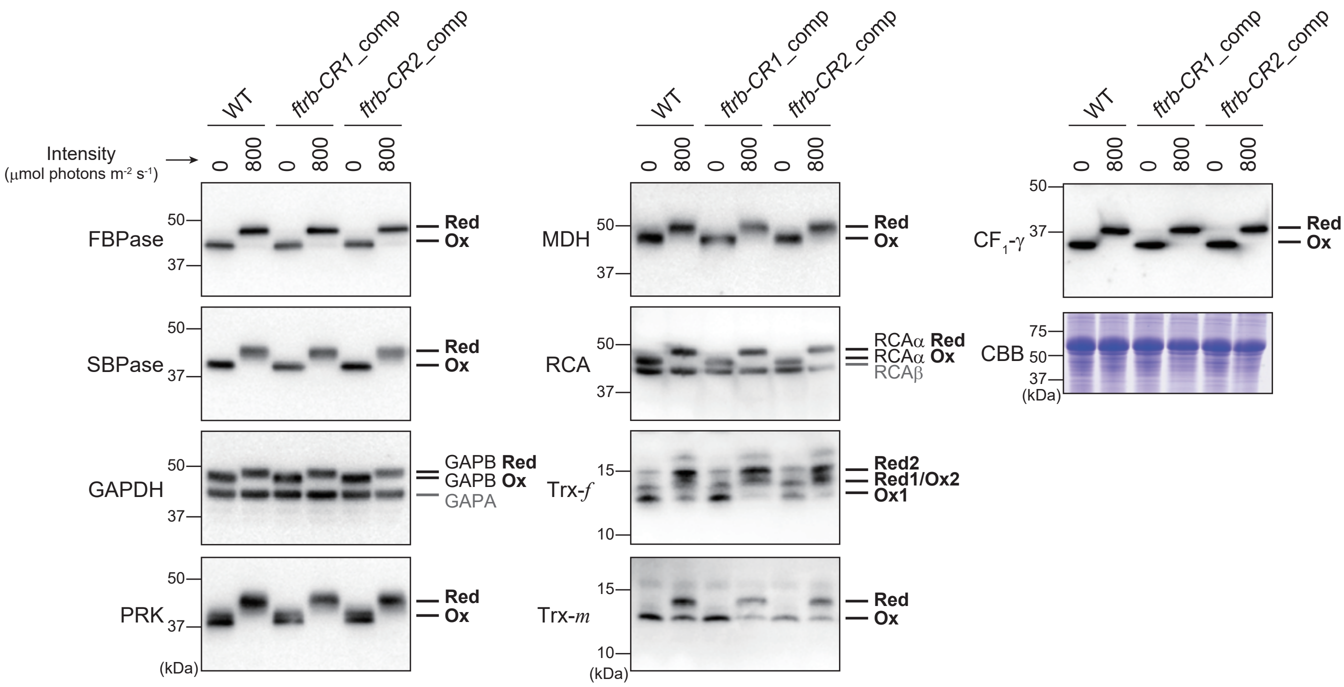

Fig. S5. Recovery of protein redox responses in *ftrb-CR*_comp plants. Plants were irradiated at 800 μmol photons m^{-2} s^{-1} for 20 min. The same amount of total leaf protein was loaded onto each lane. As a loading control, the Rubisco large subunit was stained with CBB. For GAPDH, redox-sensitive GAPB and redox-insensitive GAPA were detected. For RCA, redox-sensitive RCAα and redox-insensitive RCAβ were detected. Ox, oxidized form; Red, reduced form.

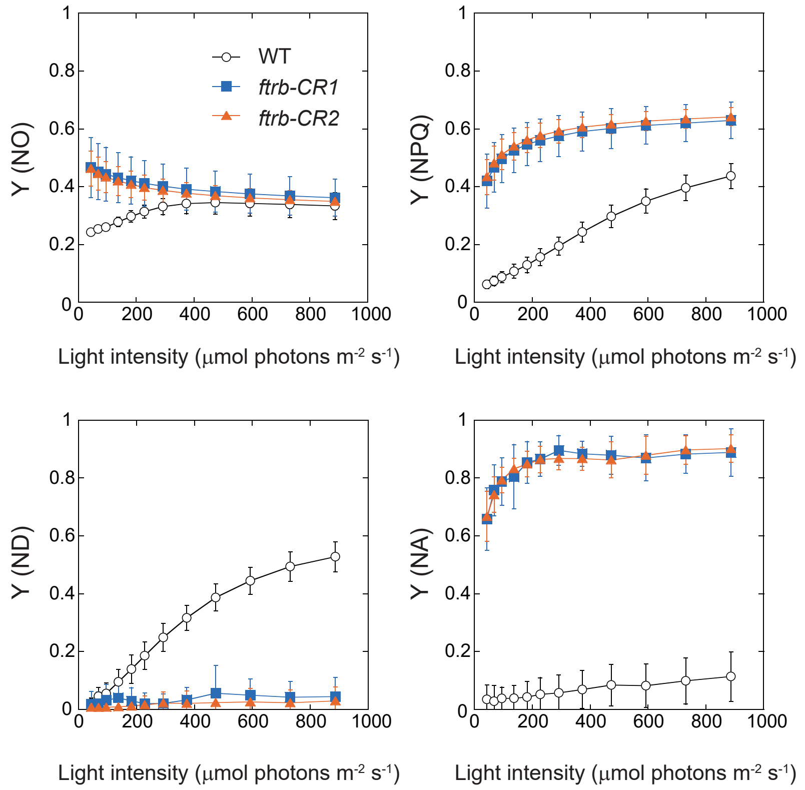

Fig. S6. Photosynthetic electron transport measurements in WT and *ftrb-CR* mutants. Several parameters including Y (NO), Y (NPQ), Y (ND), and Y (NA) were determined at several light intensities. Each value represents the mean ± SD (n = 6–7 biological replicates).

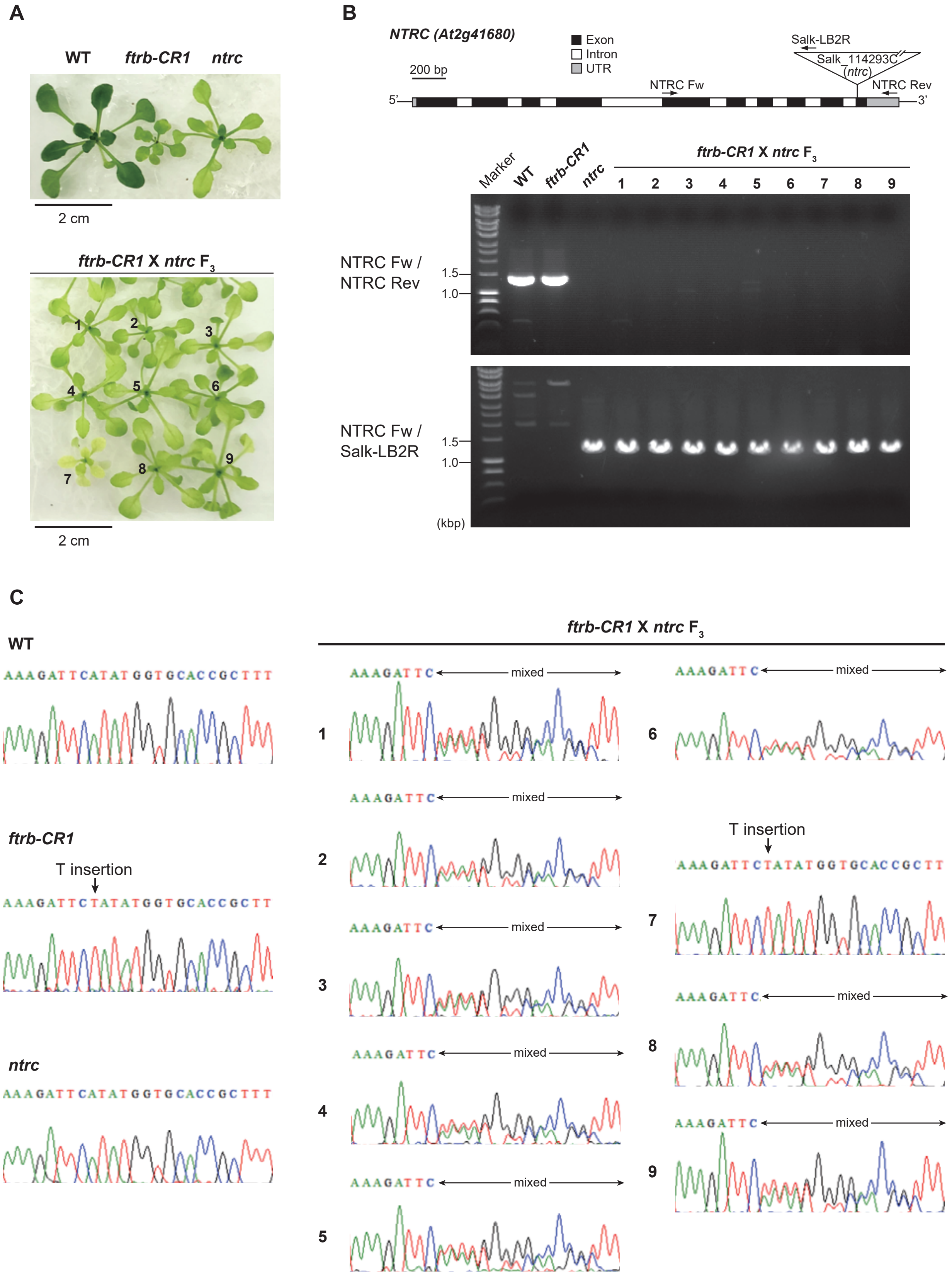

Fig. S7. Screening for the *ftrb-CR1 ntrc* double homozygous mutant. (A) Plants in the F_3 generation of *ftrb-CR1* × *ntrc* are shown with WT, *ftrb-CR1* mutant, and *ntrc* mutant. The images are identical to those in Fig. 5A. The numbers correspond to those in (B) and (C). (B) Genomic PCR for the *NTRC* gene. The simplified structure of the *NTRC* gene, T-DNA insertion site in the *ntrc* mutant, and primer binding sites are shown in the upper image. (C) DNA sequences around the Cas9-targeted site in the *FTRB* gene. Two mixed signals indicate that the genotype is heterozygous.

Table S1. Primers used in this study

Name	Sequence (5' to 3')	Purpose
FTRB sgRNA Fw	TTCGCTGAGCATAAAGATTCATA	Plasmid construction for disrupting *FTRB*
FTRB sgRNA Rev	AAACTATGAATCTTTATGCTCAG	Plasmid construction for disrupting *FTRB*
FH41	AAACGACGGCCAGTGCCAGAATTGGGCCCGACGTCG	Plasmid construction for disrupting *FTRB*
FH42	TACTGACTCGTCGGGTACCAAGCTATGCATCCAACGCG	Plasmid construction for disrupting *FTRB*
FTRB_pRI201 Fw	GTTGATACATATGAATCTTCAAGCTGTTTCTTG	Amplification of *FTRB* for plasmid consruction
FTRB_pRI201 Rev	GAATTGTCGATCACATGTTAGCTGTAGTTTC	Amplification of *FTRB* for plasmid consruction
pRI201_FTRB Fw	TAACATGTGATCGACAATTCTGAATCAACAAC	Plasmid construcion for *FTRB* expression
pRI201_FTRB Rev	GAAGATTCATATGTATCAACAGTGAAG	Plasmid construcion for *FTRB* expression
pRI201_Fw2-2	TCATTTGGAGAGAACACGGG	Screening for transformant plants
pRI201_Rev2-2	CTAGCTTTTTATTTGACACACC	Screening for transformant plants
FTRB Fw	AGTTTGAACCGATTCGATGG	Amplification of *FTRB* for sequence analysis
FTRB Rev	TTTCGTGTAAAGTTGGATCC	Amplification of *FTRB* for sequence analysis
FTRB_seq	TGGCGAGCATTGGTTTCTGG	Sequence analysis of *FTRB*
NTRC Fw	ATCAACAATCCAAACATCAC	Genomic PCR
NTRC Rev	GTTGTTTTGTAAAATCTTAAAGC	Genomic PCR
Salk-LB2R	GACCGCTTGCTGCAACTCTCTCA	Genomic PCR
